# Supplementary figures and images for: Systems-level proteomics and metabolomics reveals the diel molecular landscape of diverse kale cultivars
Source: Front Plant Sci. 2023 Jul 28;14:1170448. doi: 10.3389/fpls.2023.1170448 (PMC10421703; doi:10.3389/fpls.2023.1170448)

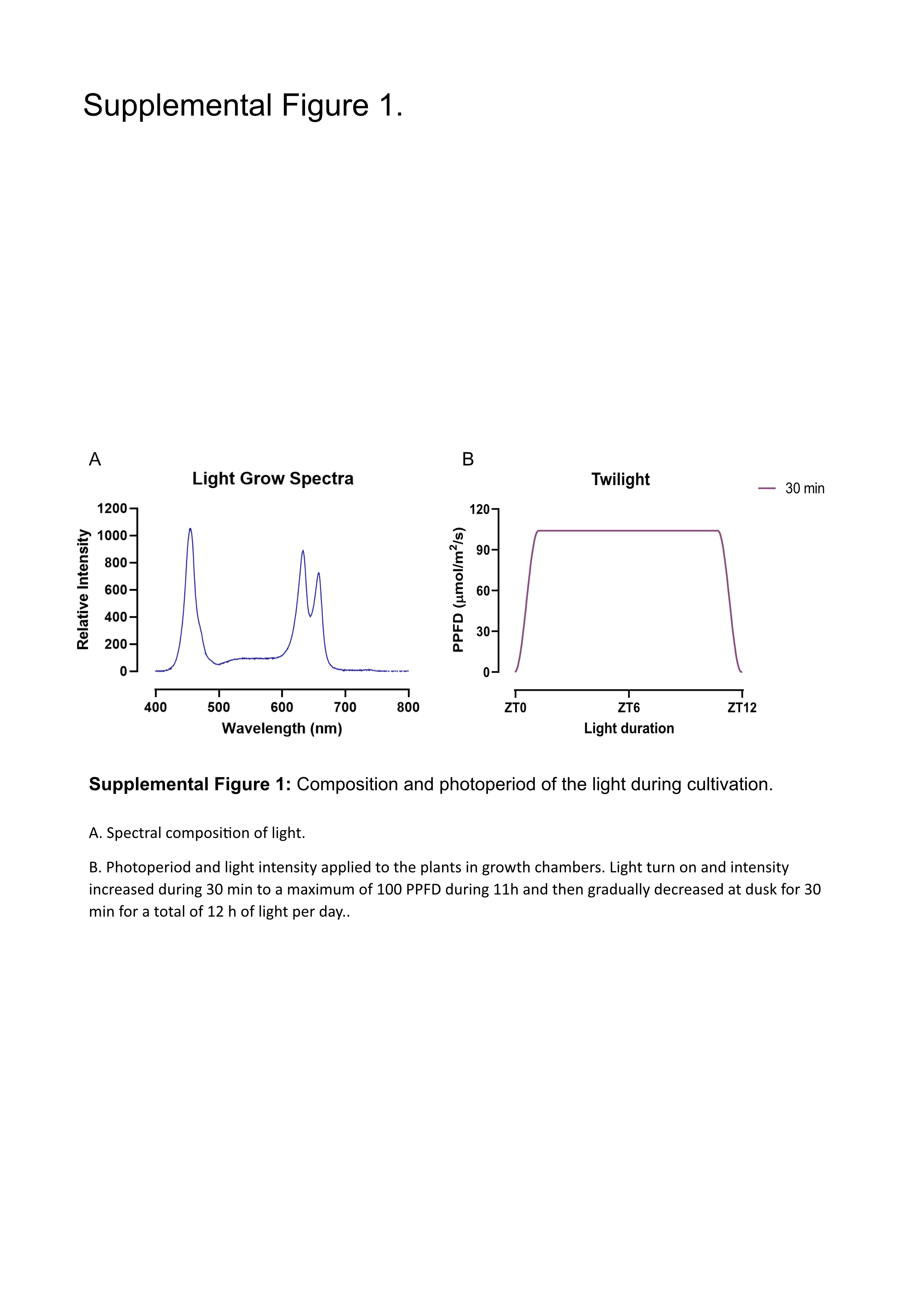

Supplement: Supplementary file 1 [file Image_1.tiff]

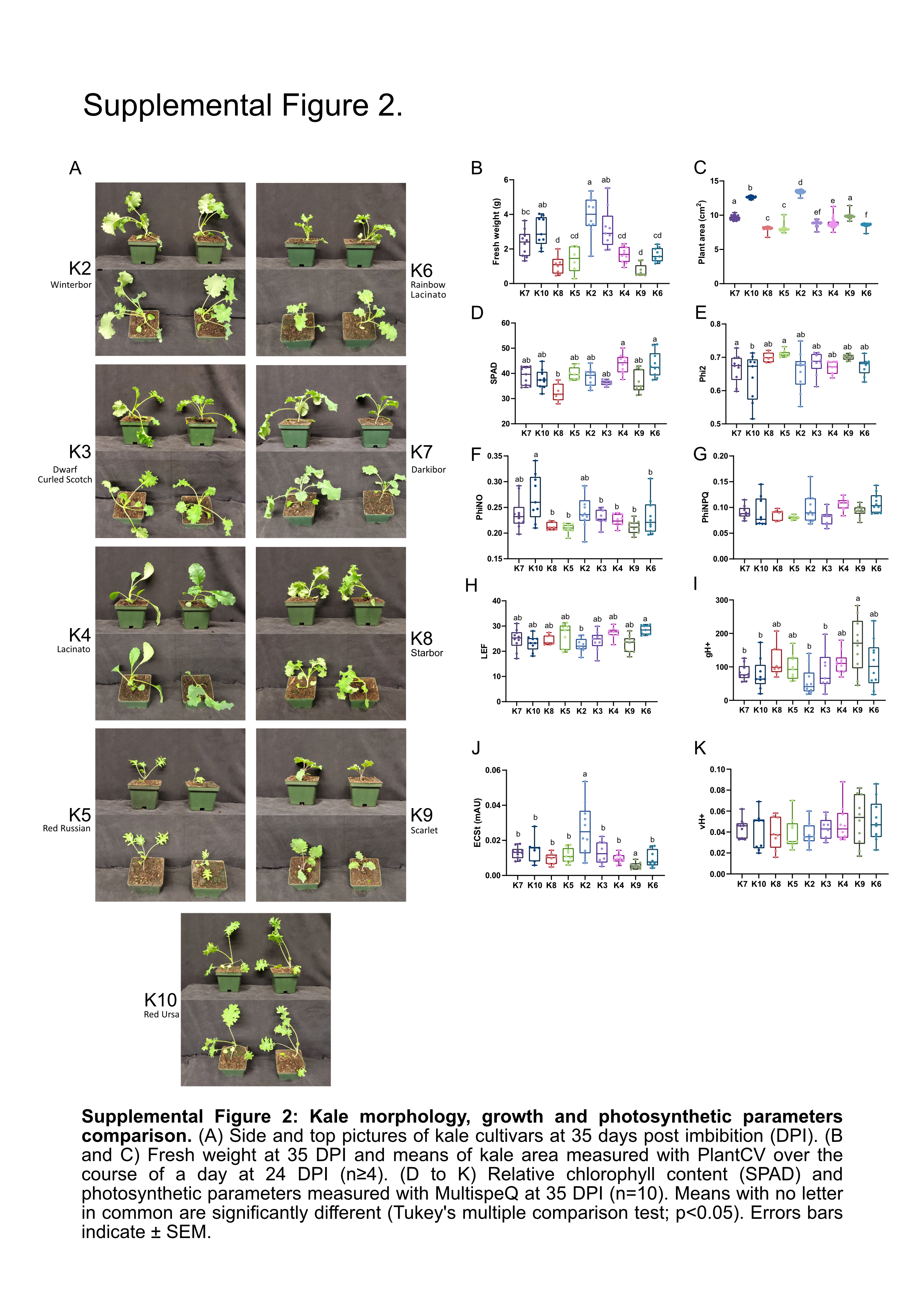

Supplement: Supplementary file 8 [file Image_2.tiff]

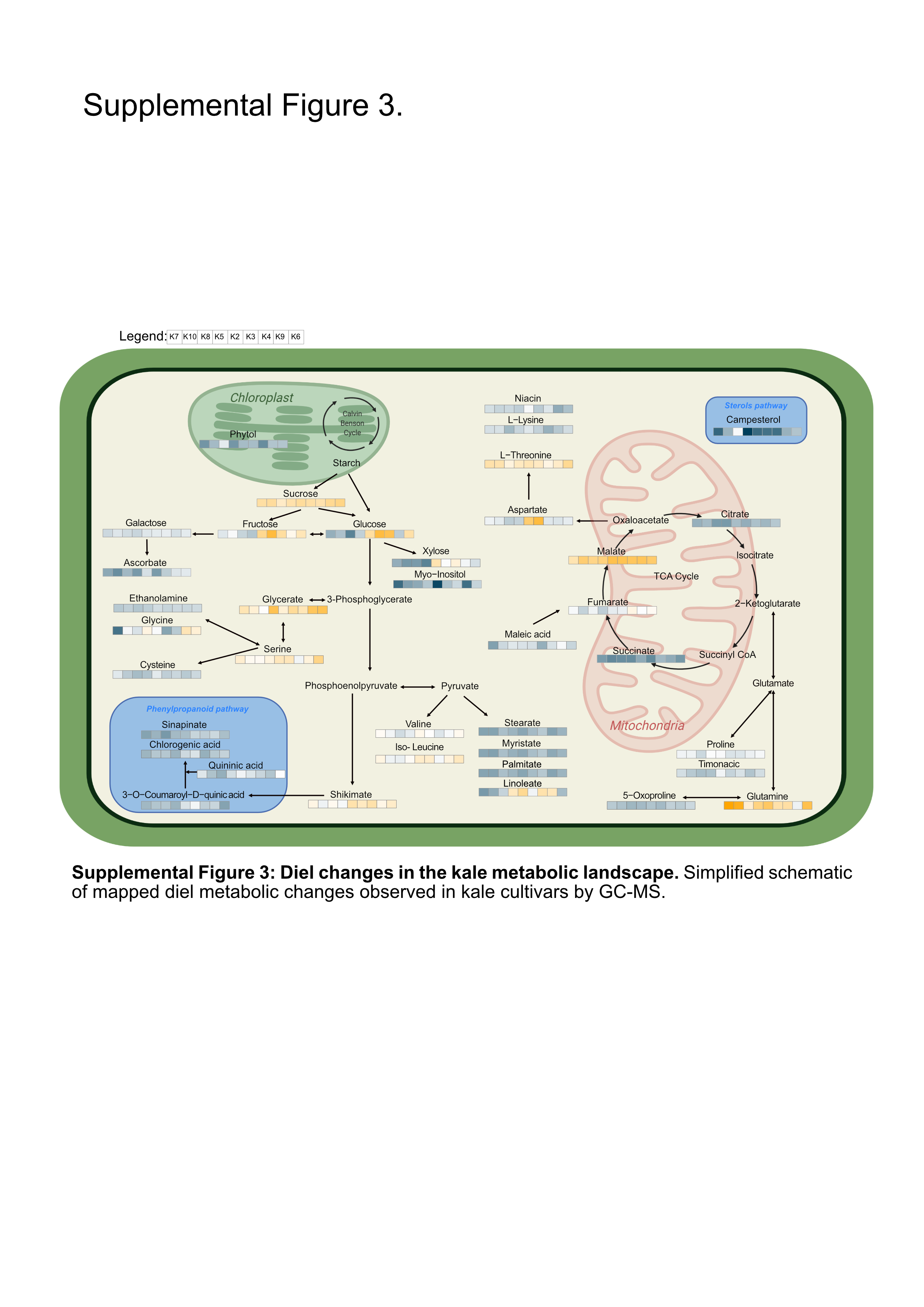

Supplement: Supplementary file 9 [file Image_3.tiff]

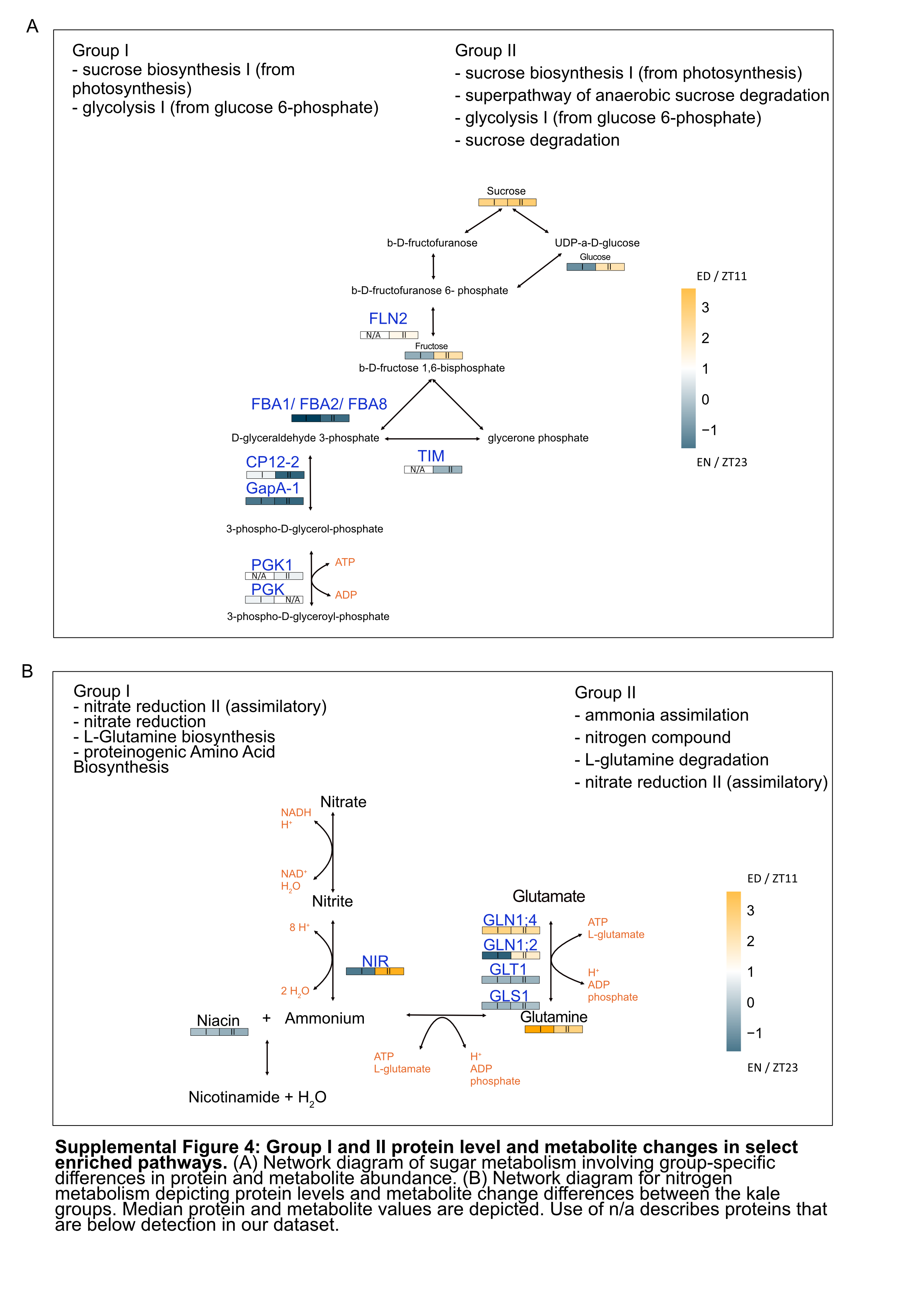

Supplement: Supplementary file 10 [file Image_4.tiff]
